# Stringent DDI-based Prediction of *H. sapiens-M. tuberculosis *H37Rv Protein-Protein Interactions

**DOI:** 10.1186/1752-0509-7-S6-S6

**Published:** 2013-12-13

**Authors:** Hufeng Zhou, Javad Rezaei, Willy Hugo, Shangzhi Gao, Jingjing Jin, Mengyuan Fan, Chern-Han Yong, Michal Wozniak, Limsoon Wong

**Affiliations:** 1 NUS Graduate School for Integrative Sciences & Engineering, National University of Singapore, Singapore; 2 School of Computing, National University of Singapore, Singapore; 3 Department of Medicine, Brigham and Women's Hospital, USA; 4 Department of Microbiology and Immunobiology, Harvard Medical School, Harvard University, USA; 5 Department of Environmental Health, Harvard School of Public Health, Harvard University, USA; 6 Faculty of Mathematics, Informatics and Mechanics, University of Warsaw, Poland

**Keywords:** protein-protein interaction (PPI), *H. sapiens-M. tuberculosis*, H37Rv PPIs, Domain-domain interaction (DDI)

## Abstract

**Background:**

*H. sapiens-M. tuberculosis *H37Rv protein-protein interaction (PPI) data are very important information to illuminate the infection mechanism of *M. tuberculosis *H37Rv. But current *H. sapiens-M. tuberculosis *H37Rv PPI data are very scarce. This seriously limits the study of the interaction between this important pathogen and its host *H. sapiens*. Computational prediction of *H. sapiens-M. tuberculosis *H37Rv PPIs is an important strategy to fill in the gap. Domain-domain interaction (DDI) based prediction is one of the frequently used computational approaches in predicting both intra-species and inter-species PPIs. However, the performance of DDI-based host-pathogen PPI prediction has been rather limited.

**Results:**

We develop a stringent DDI-based prediction approach with emphasis on (i) differences between the specific domain sequences on annotated regions of proteins under the same domain ID and (ii) calculation of the interaction strength of predicted PPIs based on the interacting residues in their interaction interfaces.

We compare our stringent DDI-based approach to a conventional DDI-based approach for predicting PPIs based on gold standard intra-species PPIs and coherent informative Gene Ontology terms assessment. The assessment results show that our stringent DDI-based approach achieves much better performance in predicting PPIs than the conventional approach. Using our stringent DDI-based approach, we have predicted a small set of reliable *H. sapiens-M. tuberculosis *H37Rv PPIs which could be very useful for a variety of related studies.

We also analyze the *H. sapiens-M. tuberculosis *H37Rv PPIs predicted by our stringent DDI-based approach using cellular compartment distribution analysis, functional category enrichment analysis and pathway enrichment analysis. The analyses support the validity of our prediction result. Also, based on an analysis of the *H. sapiens-M. tuberculosis *H37Rv PPI network predicted by our stringent DDI-based approach, we have discovered some important properties of domains involved in host-pathogen PPIs. We find that both host and pathogen proteins involved in host-pathogen PPIs tend to have more domains than proteins involved in intra-species PPIs, and these domains have more interaction partners than domains on proteins involved in intra-species PPI.

**Conclusions:**

The stringent DDI-based prediction approach reported in this work provides a stringent strategy for predicting host-pathogen PPIs. It also performs better than a conventional DDI-based approach in predicting PPIs. We have predicted a small set of accurate *H. sapiens-M. tuberculosis *H37Rv PPIs which could be very useful for a variety of related studies.

## Background

Tuberculosis is an infectious disease which causes millions of deaths each year. *M. tuberculosis*--the causative agent of tuberculosis-- infects around one-third of the world's population [1,2]. Tuberculosis is one of the most common opportunistic infections in HIV-infected patients and it is also one of the most common death causes among HIV patients [3,4].

Host-pathogen PPIs are essential for a pathogen's colonization, adhesion and invasion of host cells, which are crucial for the understanding of infection mechanism and the interaction between pathogen and host. Unfortunately, high-quality large-scale experimental host-pathogen PPIs are not available in many host-pathogen systems, especially between *H. sapiens *and *M. tuberculosis *H37Rv. Many computational approaches have been developed to predict host-pathogen PPIs including approaches based on homology, interacting domain/motif, structure, and even machine learning [5]. DDI-based approaches are often used for predicting both intra-species and inter-species PPIs, with the assumption that domain-domain interactions mediate the protein-protein interactions, because domains are the basic building blocks determining the structure and function of proteins [5].

In this work, we develop a stringent DDI-based approach for predicting the *H. sapiens-M. tuberculosis *H37Rv PPIs by taking into account of the differences between each specific domain sequence (we name it "domain instance") on each annotated region of proteins under the same domain ID. The interactions between query domain instances are made based on very stringent sequence alignment to the structural template domain instances. Moreover, we adopt an effective scoring strategy in ranking how likely the predicted proteins are interacting with each other by examining the interacting residues in the interaction interfaces. As long as the two amino acids have one of the atomic interaction: hydrogen bonds, electrostatic or van de Waals interactions between two domain instances that are defined as interacting residues in this study. Thus, we are standing on a much more stringent and finer level of domain interaction by examining not only the sequence similarity of each domain instances but also the interaction interface compatibility between them. In contrast, conventional DDI-based approaches generally use some popular tools to annotate the domains in proteins and then see whether two proteins contain a pair of domains whose IDs match a pair of domains that are known to interact in some other pair of proteins. Matching query domain instance to template domain instance based on domain ID--as done in such conventional DDI-based approaches--is rather coarse and often leads to matching of domain instances that do not have the same interaction interfaces.

Using gold standard *H. sapiens *PPIs, we assess the performance of our stringent DDI-based approach and the conventional DDI-based approach by comparing their precision-recall curves and the number of predicted PPIs overlapping with gold standard PPIs. We also use the percentage of coherent informative Gene Ontology(GO) annotations to assess the predicted *H. sapiens *PPIs to compare the performance of our stringent DDI-based approach and the conventional DDI-based approach. These assessments demonstrate that our stringent DDI-based approach has much better performance than a conventional DDI-based approach. Cellular compartment distribution analysis, pathway enrichment analysis, and functional category enrichment analysis supports the validity of our predicted *H. sapiens-M. tuberculosis *H37Rv PPI dataset. Our stringent DDI-based approach can be used for predicting host-pathogen PPIs in a variety of different host-pathogen systems. We have also discovered some interesting properties of both pathogen and host proteins participating in host-pathogen PPIs, including the tendency to have more domains, and the domains on the proteins involved in host-pathogen PPIs tend to have much higher degrees.

## Methods

Our stringent DDI-based approach predicts PPIs by inferring domain instance interactions from structural template domain instance interactions. Using the MUSCLE alignment program [6], we accurately align query protein domain instances to template domain instances using a stringent threshold(length difference *≤ *20% and sequence similarity *≥ *50%) and transfer the possible interactions between template structural domain instances to our query domain instances. Here the length difference is calculated by the difference of length (longer sequence length minus shorter sequence length) divided by the length of query domain instance; sequence similarity is the number of correctly aligned residues divided by the length of query domain instance.

We then predict the possible PPIs from interacting query domain instances. The structural domain instances are extracted from the 3did database [7]. Each interacting query domain instance pair is scored according to the similarity of the interaction residues in the interaction interfaces, and the best query instance score is used to represent the interaction strength of the predicted PPI (how likely the two proteins in the PPI are interacting each with other). We predict both host-pathogen (*H. sapiens-M. tuberculosis *H37Rv) and intra-species (*H. sapiens*) PPIs in this work. For a comparison study, we use a conventional DDI-based approach [8] to predict possible intra-species (*H. sapiens*) PPIs. We assess our stringent DDI-based approach and the conventional approach using gold standard *H. sapiens *PPIs and by the percentage of the predicted PPIs that have coherent informative GO annotation. These assessments show that our stringent DDI-based approach has better performance in predicting PPIs than the conventional approach. Cellular compartment distribution analysis, pathway enrichment analysis, and functional enrichment analysis support our prediction results and show that the predicted PPIs correspond to the *M. tuberculosis *H37Rv infection process. We further analyze some of the basic domain properties of proteins involved in the host-pathogen Protein-Protein Interaction Network (PPIN), comparing with other proteins involved in intra-species PPIN, by examining the number of domains and domain interaction degrees.

### PPI prediction--our stringent DDI-based approach

It is a reasonable assumption that an observed interaction between two domain instances can be used to infer the interaction of another domain instance pair, provided the two domain instance pairs are sufficiently similar as to preserve the relevant interaction interfaces. Specifically, consider two protein domains A and B. Let *A_i _*and *B_i _*be two instances of domain A and B, respectively. Suppose we know that these two instances have a direct physical interaction (from the crystal structure of a protein complex). Given the observation of *A_i _*and *B_i_*, one could infer the interaction of another instance pair of A and B, *A_j _*and *B_j_*, by using a sequence similarity threshold between (*A_i_, B_i_*) and (*A_j_, B_j_*).

In general, conventional DDI-based approaches disregard the details of the interaction between these domain instances in the real 3D space--i.e., the interaction interface between the two instances--and thus effectively match the domain instances based on name. In contrast, we formulate a stringent approach that emphasizes the similarity of the interaction interface of the domain instances. Specifically, we assign a positive prediction score on pairs with high interface residue similarity with respect to the observed interaction instances in the existing protein structural data.

The data on structural domain instances, including the interacting domain pair, the structural and sequence details of interacting domain instances, the interacting residues in the interaction interfaces are extracted from the 3did database [7]. These individual domain instances with 3did structural data serve as "template domain instances", and pairs of interacting domain instances with 3did structural data serve as "template interacting domain instance pairs". The fasta sequences of all *H. sapiens *and *M. tuberculosis *H37Rv proteins are obtained from Uniprot [9]. Their respective protein domain annotations are obtained from InterPro [10], from which we collect the sequences of domain instances which have at least one template domain instance from 3did. These domain instances are named the "query domain instances". They are aligned to each of the template domain instances under the same domain ID using the MUSCLE alignment program [6]. Only query domain instances meeting the stringent threshold of length difference *≤ *20% and sequence similarity*≥ *50% are kept for the following analysis. For each pair (*A_i_, B_i_*) of query domain instances that meets the stringent alignment threshold to a template interacting domain instance pair (A, B), we infer the interaction interface residues in (*A_i_, B_i_*) as the residues that are aligned to the interaction interface residues in (A, B). A score of this interaction interface of (*A_i_, B_i_*) is then computed by summing the BLOSUM62 substitution score [11] between the residues in this interaction interface and the corresponding residues in the interaction interface of (A, B) that they are aligned to. This score is defined as the "domain instance interaction strength". Query domain instances with multiple possible template instances are scored based on the template with the best domain instance interaction strength. For any possible pair of proteins, if they have a query domain instance pair (one domain instance on each of the two proteins), then these two proteins are predicted to be interacting with an interaction score equaling the domain instance interaction strength of that query domain instance pair. If the protein pair has more than one underlying query domain instance interaction pair, then the query domain instance pair with the best score is used to represent the protein pair. This best score is taken as "interaction strength" of this protein pair.

**Figure 1:**
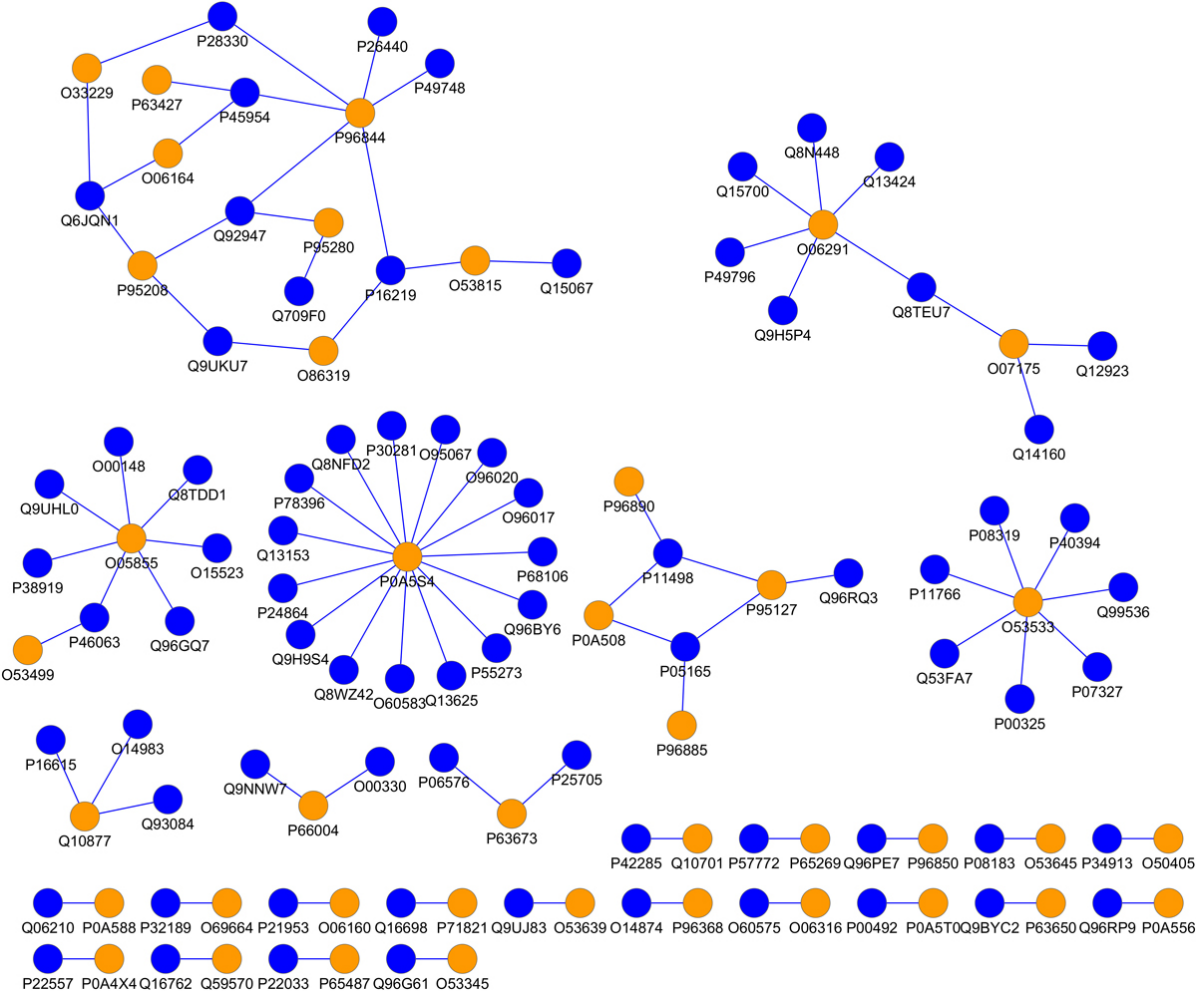
**Visualization of the predicted *H. sapiens-M. tuberculosis *H37Rv PPI network. The orange dots are *M. tuberculosis *H37Rv proteins, while the blue dots are *H. sapiens *proteins**.

We apply this DDI-based prediction approach on human proteins and this results in 839 predicted human intra-species PPIs. We also predict inter-species PPIs (*H. sapiens-M. tuberculosis *H37Rv) to identify a set of potential host-pathogen PPIs; the result is visualized in Figure 1.

### PPI prediction--a conventional DDI-based approach

The conventional DDI-based approach predicts how likely two proteins are interacting with each other by integrating known intra-species PPIs with domain profiles based on an association method (sequence-signature algorithm) proposed by Sprinzak *et al*. [12] Specifically, domains are annotated in each protein in a known intra-species PPI dataset. Then, the probability *P*(*d, e*) that two proteins containing a specific pair of domains (*d, e*) would interact is estimated for each pair of domains in a Bayesian manner. Finally, given a new pair of proteins, their probability of interaction is estimated by a naive combination $\left(=\;1-\prod_{i}\prod_{j}\left(1-P\left(d_{i},e_{j}\right)\right)\right)$ of the probabilities from each pair of domains (*d_i_, e_j_*) contained in the pair of proteins [8]. This predicted probability(called "interaction strength" of the conventional approach) can be used to rank the list of predicted PPIs.

This conventional DDI-based approach is applied to predict host-pathogen PPIs as follows. For each pair of proteins (one in *H. sapiens *and one in *M. tuberculosis*), we compute their probability of interactions as described above based on DDIs in a yeast physical PPI dataset collected from MINT [13], BioGRID [14], and IntAct [15]. This conventional DDI-based approach is also applied to predict human intra-species PPIs. In this case, for each pair of proteins (both in *H. sapiens*), we compute their probability of interactions as described above based on DDIs in the same yeast physical PPI dataset. As a control study, we ensure that the domains considered are the same domain set considered in the stringent DDI-based approach--i.e., we restrict the domain set to domains contained in 3did.

### Assessment based on gold standard *H. sapiens *PPIs

**Figure 2:**
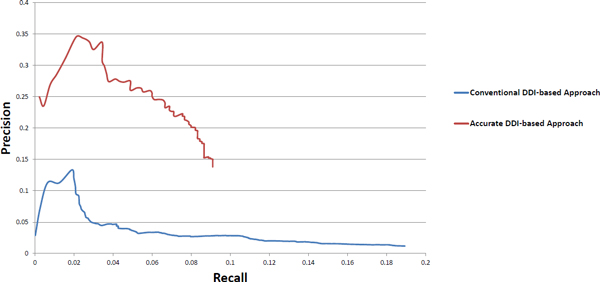
**Assessment of the stringent and the conventional DDI-based approaches through gold standard *H. sapiens *PPIs**.

Because no large-scale high-quality *H. sapiens-M. tuberculosis *PPI dataset is currently available, we can only assess the performance of the stringent and the conventional DDI-based approaches in a intra-species system. We use both the stringent and the conventional DDI-based approach to predict possible *H. sapiens *PPIs and assess the predicted PPI datasets using gold standard *H. sapiens *PPIs. The gold standard *H. sapiens *PPIs are the physical PPIs collected from MINT [13], BioGRID [14], and IntAct [15]. We sort the predicted *H. sapiens *PPIs according to their predicted "interaction strength" in the respective DDI-based approaches, and compare the top PPIs with the gold standard *H. sapiens *PPIs. For the stringent DDI-based approach, we sort the prediction results and iterate 10 PPIs at a time--which means the first time we choose all the top 839 PPIs, the second time we choose the top 829 PPIs, etc.--and then we compare with the gold standard *sapiens *PPIs to calculate the precision and recall and plot the precision-recall curve. The precision-recall curve of the conventional DDI-based approach is plotted in the same way. The precision-recall curves are plotted together for a better comparison in Figure 2.

As the two PPI datasets predicted by the stringent and the conventional DDI-based approaches are very different in the number of PPIs, their precision-recall curves may not be sufficient for judging the performance of the two prediction approaches. So we choose some special points to provide a more informative statistics. The stringent DDI-based approach predicted 839 *H. sapiens *PPIs and 82 of which overlap with the gold standard PPIs. We consider a similar amount of conventional-approach predicted *H. sapiens *PPIs(top 885 PPIs), and see how many of these predicted PPIs overlap with gold standard. We also choose another point on the precision-recall curve, that has a similar number of overlapping PPIs with the gold standard as the stringent DDI-based approach, and see how many predictions are made by conventional DDI-based approach. The results are shown in Table 1.

**Table 1:** Assessment of the stringent and the conventional DDI-based approaches through gold standard *H. sapiens *PPIs.

Conventional DDI-based Approach	Overlap with Gold Standard
Top 3085 PPIs	81
Top 885 PPIs	11

**Stringent DDI-based Approach**	**Overlap with Gold Standard**

All 839 PPIs	82

This table summarizes the assessment of the stringent and the conventional DDI-based approaches through gold standard human PPIs. In order for the conventional DDI-based approach to attain an amount of overlap with gold standard human PPIs similar to the stringent DDI-based approach, a much larger number of (false positive) predicted PPIs must be accepted. Conversely, if the conventional DDI-based approach is restricted to a similar number of predictions as the stringent DDI-based approach, a much lower overlap with gold standard human PPIs must be accepted.

### Assessment using coherent informative GO annotation of predicted *H. sapiens *PPIs

**Figure 3:**
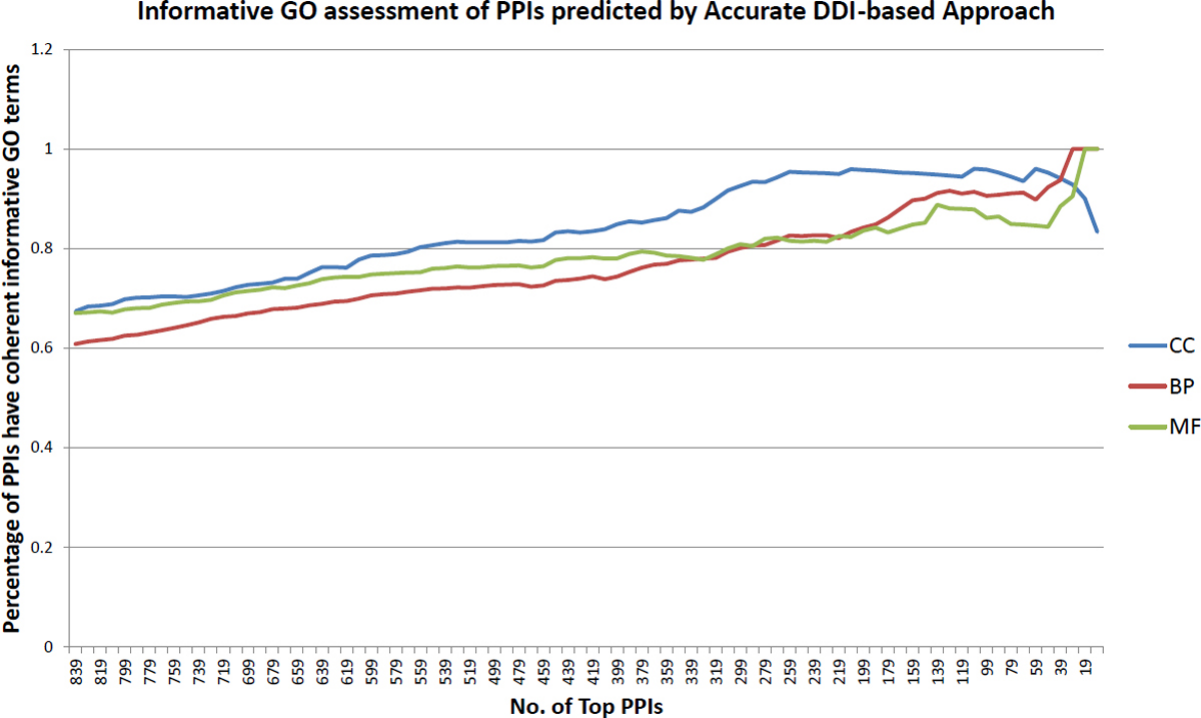
**Informative GO assessment of the PPIs predicted by the stringent DDI-based approach**.

**Figure 4:**
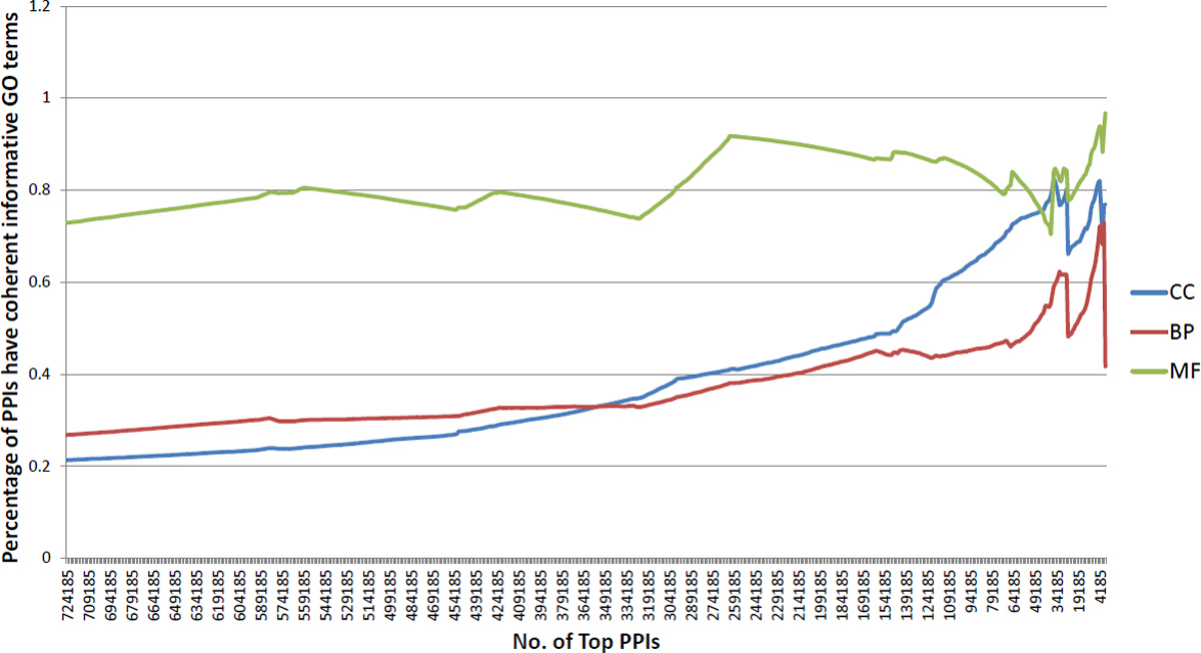
**Informative GO assessment of the PPIs predicted by the conventional DDI-based approach**.

**Figure 5:**
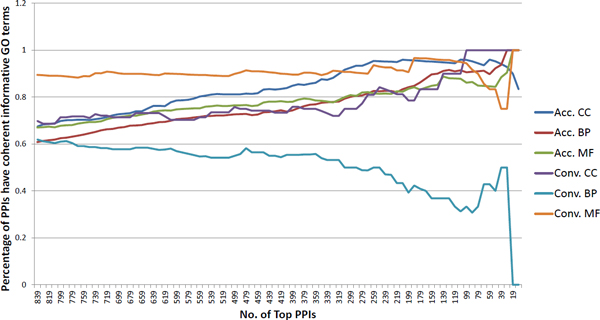
**Informative GO assessment of the top 839 PPIs predicted by the stringent and the conventional DDI-based approaches**. "Acc." means the PPIs predicted by the stringent DDI-based approach; "Conv." means the PPIs predicted by the conventional DDI-based approach.

A PPI is more likely to be real, if its two protein components have coherent GO annotation--i.e., the two proteins are annotated with at least one "informative" GO term in common. The percentage of PPIs having coherent GO annotation is also frequently used in assessing the quality of the PPI dataset [16]. Note that GO contains three hierarchical ontologies, and terms at the root level have more proteins annotated with them, while terms at the leaf level have fewer proteins annotated with them. In order to avoid bias, we only keep informative GO terms for the assessment here. An informative GO term is defined as a GO term that has at least 30 proteins annotated with it but each of its child terms has fewer than 30 proteins annotated with it. This definition of informative GO term is also used in another work [16] for assessing PPI dataset quality in *M. tuberculosis *H37Rv. For the PPI datasets predicted by the stringent DDI-based approach and by the conventional DDI-based approach, the PPIs in each dataset are sorted according to their respective "interaction strength" (which is an indicator of how likely the PPIs are real), then the percentage of PPIs that has coherent informative GO terms are calculated. For each dataset we move along from the bottom to the top to set the threshold of how many top PPIs are considered, and calculate the percentage of these PPIs having coherent informative GO terms. For the stringent DDI-based approach we choose an interval of 10 PPIs and move along from the bottom to the top(e.g. top 839 PPIs, top 829 PPIs, etc.), then calculate the percentage of PPIs that have coherent informative GO terms and plot the percentage; see Figure 3. For the conventional DDI-based approach, we plot the percentage in the same way; but as the conventional DDI-based approach predicts much more PPIs, we choose interval of 1000 PPIs while making the plot; see Figure 4. To better compare and assess the performance of the stringent and the conventional DDI-based approaches, we focus on the top 839 PPIs predicted by both approaches, choosing interval of 10 PPIs as we plot the percentage of PPIs having coherent GO annotation on the same figure; see Figure 5. When assessing the quality of two PPI datasets based on informative GO terms, the number of GO terms that are annotated to the proteins of that PPI dataset also influences the percentage of PPIs having coherent informative GO terms in that dataset. Therefore we summarize the number of informative GO terms in the 839 PPIs predicted by the stringent DDI-based approach, and the number of informative GO terms in the 724185 PPIs and in the top 839 PPIs predicted by the conventional DDI-based approach; see Table 2. All these analysis results support the conclusion that our stringent DDI-based approach is better at predicting reliable PPIs especially when it comes to a small-scale dataset.

**Table 2:** Number of informative GO terms annotated to proteins involved in PPIs predicted by the stringent and the conventional DDI-based approach.

Conventional DDI-based Approach	CC term No.	BP term No.	MF term No.
All 724185 PPIs	140	880	247
Top 839 PPIs	28	94	34
**Stringent DDI-based Approach**	**CC term No.**	**BP term No.**	**MF term No.**
All 839 PPIs	116	820	237

This table summarizes the number of informative GO terms annotated to proteins involved in PPIs predicted by the stringent and the conventional DDI-based approach.

### Cellular compartment distribution of *H. sapiens *proteins targeted by the predicted host-pathogen PP

The assessments above prove that our stringent DDI-based approach has a much better performance than the conventional DDI-based approach in predicting more reliable intra-species PPIs. We now analyze the host-pathogen PPIs predicted by our stringent DDI-based approach.

The cellular compartments of the *H. sapiens *proteins targeted by the predicted *H. sapiens-M. tuberculosis *H37Rv PPIs are useful in telling the quality of the predicted host-pathogen PPIs. If the targeted *H. sapiens *proteins are located in cellular compartments that are very relevant to the pathogen's infection or are very likely to be involved in interactions with the pathogen, then the result supports the host-pathogen predictions. Gene Ontology (Cellular Compartment, CC) is a very comprehensive annotation system for human proteins. However, as the Gene Ontology is hierarchical, we only use informative CC terms for our analysis.

**Figure 6:**
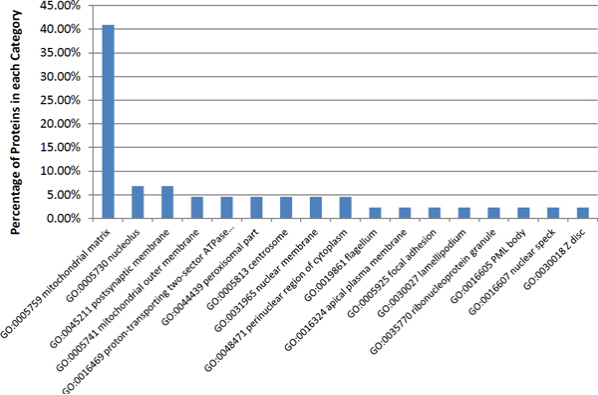
**Cellular compartment distribution of *H. sapiens *proteins targeted by host-pathogen PPIs predicted by the stringent DDI-based approach**.

Different from using the coherent informative GO annotation for the assessment of the human intra-species PPI dataset, we choose a different resolution of the GO terms for the category distribution analysis of human proteins involved in *H. sapiens-M. tuberculosis *PPIs: An informative CC term is defined here to be a term that has at least 90 proteins annotated with it, but each of its child terms has less than 90 proteins annotate with it. The cellular compartment distribution tells how many proteins(and the percentage) in the datasets fall into each cellular compartment. We show the cellular compartments of the *H. sapiens *proteins that are targeted by the stringent DDI-based prediction approach in Table 3 and Figure 6.

**Table 3:** Cellular compartment distribution of *H. sapiens *proteins targeted by host-pathogen PPIs predicted by the stringent DDI-based approach.

Cellular Compartment	Percentage(%)	No. of Proteins
GO:0005759 mitochondrial matrix	40.91%	18
GO:0005730 nucleolus	6.82%	3
GO:0045211 postsynaptic membrane	6.82%	3
GO:0005741 mitochondrial outer membrane	4.55%	2
GO:0016469 proton-transporting two-sector ATPase complex	4.55%	2
GO:0044439 peroxisomal part	4.55%	2
GO:0005813 centrosome	4.55%	2
GO:0031965 nuclear membrane	4.55%	2
GO:0048471 perinuclear region of cytoplasm	4.55%	2
GO:0019861 flagellum	2.27%	1
GO:0016324 apical plasma membrane	2.27%	1
GO:0005925 focal adhesion	2.27%	1
GO:0030027 lamellipodium	2.27%	1
GO:0035770 ribonucleoprotein granule	2.27%	1
GO:0016605 PML body	2.27%	1
GO:0016607 nuclear speck	2.27%	1
GO:0030018 Z disc	2.27%	1

This table summarizes cellular compartment distribution of *H. sapiens *proteins targeted by host-pathogen PPIs predicted by the stringent DDI-based approach.

### Functional enrichment analysis of proteins involved in host-pathogen PPIs

Functional enrichment analysis is important for revealing the functional relevance of the proteins involved in the host-pathogen PPIs predicted by our stringent DDI-based approach. The presence of enriched(over-represented) functional categories that are closely related to pathogen infection, serves as a support for the validity of the predicted host-pathogen PPIs. The Gene Ontology (Molecular Function, MF) is a comprehensive functional annotation system. Therefore we conduct MF term enrichment analysis on the *H. sapiens *proteins involved in the *H. sapiens-M. tuberculosis *H37Rv PPIs predicted by our stringent DDI-based approach. We use the DAVID database [17] for the GO term enrichment analysis. Results are shown in Table 4 (significantly enriched level 5 MF terms, threshold "count *>*2, p-value *<*0.1"). On the other hand, as we have found in another work [16], most of the GO annotations for *M. tuberculosis *H37Rv are not specific enough to provide effective functional enrichment analysis. Thus, the functional analysis of *M. tuberculosis *H37Rv proteins are not discussed in this work.

**Table 4:** Functional enrichment analysis of *H. sapiens *proteins involved in the host-pathogen PPI dataset predicted by the stringent DDI-based approach.

**GO terms**	**p-value**
GO:0050660 FAD binding	2.27E-11
GO:0016462 pyrophosphatase activity	3.64E-06
GO:0004022 alcohol dehydrogenase (NAD) activity	8.70E-06
GO:0032559 adenyl ribonucleotide binding	9.27E-05
GO:0042626 ATPase activity, coupled to transmembrane movement of substances	6.54E-04
GO:0015405 P-P-bond-hydrolysis-driven transmembrane transporter activity	1.09E-03
GO:0042625 ATPase activity, coupled to transmembrane movement of ions	1.27E-03
GO:0000287 magnesium ion binding	8.04E-03
GO:0004466 long-chain-acyl-CoA dehydrogenase activity	1.28E-02
GO:0003960 NADPH:quinone reductase activity	2.55E-02
GO:0070402 NADPH binding	2.55E-02
GO:0004745 retinol dehydrogenase activity	6.25E-02
GO:0019841 retinol binding	7.45E-02
GO:0042288 MHC class I protein binding	9.81E-02

This table summarizes the significantly enriched level 5 MF (Molecular Function) GO terms for *H. sapiens *proteins involved in the host-pathogen PPI dataset predicted by the stringent DDI-based approach. The analysis is produced using the DAVID database (threshold "count *>*2, p-value *<*0.1").

### Pathway enrichment analysis of proteins involved in host-pathogen PPIs

Pathway data are very important functional information for identifying a list of proteins' overall related functions in a cell. For a set of proteins which is significantly enriched in some pathways, it is very likely that this set of proteins play similar or co-ordinated roles *in vivo*. Thus, pathway enrichment analysis is also one of the most frequently used strategy for analyzing predicted host-pathogen PPIs.

We use the IntPath [18] database for the pathway enrichment analysis. IntPath is currently one of the most comprehensive integrated pathway databases. The "Identify Pathways" function in IntPath can identify the pathway enrichment of an input gene list. The "Identify Pathways" function in IntPath [18] adopts the hypergeometric test to identify the input gene list's over-representation(enrichment) in the pathways. For the *H. sapiens *protein set predicted by the stringent DDI-based approach, the pathway enrichment analysis result is shown in Table 5.

**Table 5:** Pathway enrichment analyses of *H. sapiens *proteins involved in the host-pathogen PPI dataset predicted by the stringent DDI-based approach.

Pathway names	p-value
Metabolic Pathways	4.82E-24
Fatty Acid Metabolism	4.04E-21
Valine, Leucine and Isoleucine Degradation	7.90E-19
Fatty Acid Beta Oxidation	5.00E-11
Glycolysis and Gluconeogenesis	4.84E-10
2-Oxobutanoate Degradation I	8.42E-10
p53 Signaling Pathway	3.86E-09
Ethanol Degradation II (cytosol)	5.92E-09

This Table shows the 8 most significantly enriched pathways for *H. sapiens *proteins involved in the host-pathogen PPI dataset predicted by our stringent DDI-based approach.

We also analyze the pathway enrichments for the *M. tuberculosis *H37Rv proteins, because IntPath [18] also supports pathway analysis for this and other important pathogens. The pathway analysis on the *M. tuberculosis *H37Rv proteins involved in *H. sapiens-M. tuberculosis *H37Rv PPIs predicted by the stringent DDI-based approach is given in Table 6.

**Table 6:** Pathway enrichment analyses of *M. tuberculosis *H37Rv proteins involved in the host-pathogen PPI dataset predicted by the stringent DDI-based approach.

Pathway names	p-value
Fatty Acid *β *oxidation I	6.78E-3
Naphthalene degradation	7.29E-3

This table summarizes the most significantly enriched pathways for *M. tuberculosis *H37Rv proteins involved in the host-pathogen PPI dataset predicted by our stringent DDI-based approach.

### Analysis of domain properties of proteins involved in host-pathogen PPIs

The analysis of protein domain properties considers the number of domains and the degrees of domains on proteins. The protein domain properties directly reflect differences between the proteins involved in inter-species host-pathogen PPIN and intra-species PPIN. We analyze the domain properties of both *M. tuberculosis *H37Rv and *H. sapiens *involved in the predicted host-pathogen PPIs, and comparing them with other proteins in their own intra-species PPIN. As a control experiment, we also conduct the same analysis on the *H. sapiens *proteins in the gold standard *H. sapiens*-HIV PPIs [19] to see whether the *H. sapiens *proteins in the gold standard *H. sapiens*-HIV PPIs exhibit similar properties.

As the host-pathogen PPIs are predicted by the stringent DDI-based approach, to avoid biased analysis, we use a different domain annotation system in this analysis. The annotation of both *M. tuberculosis *H37Rv and *H. sapiens *protein domains is accomplished using HMMER-V3.0 [20]. The domain profiles used in the protein domain annotation are Pfam-A [21]. The threshold for the domain annotation is E-value(iE-value) *≤ E − *20 and accuracy *≥ *0.9. For each domain annotated on each protein, we retrieve the sequences of these domains on every protein for the following analysis.

For the domain degree analysis, we obtain the DDI(Domain-Domain Interaction) data from the DOMINE database. DDIs "inferred from PDB entries" and "high confidence predictions" in the DOMINE database are considered in this study, while "medium confidence predictions" and "low confidence predictions" are discarded. For each domain, we count the number of interaction partners in the DOMINE database(only "inferred from PDB entries" and "high confidence predictions") as the degree of that domain. We analyze the above protein domain properties and summarize the results in Table 7.

**Table 7:** Protein domain property analysis result.

Organism	*H. sapiens *proteins		*H. sapiens *proteins	
**PPIN**	**Hum-Mtb**	**Hum-Hum**	**Hum-HIV**	**Hum-Hum**

Average No. of domains	1.79	1.31	1.42	1.27
P-value	4.40E-5		9.14E-17	

Average Domain degrees	17.95	10.22	13.23	9.21
P-value	1.79E-2		1.04E-10	

This table summarizes the protein domain analysis for *H. sapiens *proteins involved in the host-pathogen PPI dataset predicted by our stringent DDI-based approach comparing with the proteins involved in intra-species PPIN. Protein domain property analysis for *H. sapiens *proteins involved in gold standard *H. sapiens*-HIV PPI dataset [19] has also been conducted. In the table there are some abbreviations. Hum-Mtb: in predicted *H. sapiens-M. tuberculosis *H37Rv PPIN. Hum-Hum: in *H. sapiens *intra-species PPIN. Hum-HIV: in gold standard *H. sapiens*-HIV PPIN.

### Software Packages and Datasets

The software packages and database tools used in this study are:

• IntPath [18]

• Cytoscape [22]

• InterPro [10]

• InterProScan [23]

• DAVID [17]

The datasets used in this study are:

• *M. tuberculosis *H37Rv PPI dataset consisting of four reliable subsets of the B2H PPI dataset and STRING PPI dataset(threshold at 770) [16].

• *H. sapiens *physical PPI dataset collected from MINT [13], BioGRID [14], and IntAct [15]; date of download is November 10, 2011.

• *S. cerevisiae *physical PPI dataset collected from MINT [13], BioGRID [14], and IntAct [15]; date of download is November 10, 2011.

• Protein domain annotation (protein2ipr) from InterPro [10]; date of download is March 5th, 2012.

• DDI data from the 3did database [7](version November 28, 2010).

• DDI data from the DOMINE database V2.0 [24].

• Pfam-A Domain profiles [21].

• *H. sapiens*-HIV-1 PPI dataset downloaded from "HIV-1, human protein interaction database at NCBI" [19].

## Results

### Prediction of host-pathogen PPIs

Because of the stringent alignment threshold used for identifying query and template domain instances, lots of instances with large sequence variation under the same domain ID are filtered out, leaving very few domain instances for study. Also, our template interacting domain instances are from structurally resolved data in 3did, therefore the template domain instances are a relatively small number. Due to these two factors, our stringent DDI-based approach predicted PPI datasets are usually small. We have predicted 92 *H. sapiens-M. tuberculosis *H37Rv PPIs and this small set of predicted host-pathogen PPIs are analyzed using several approaches as discussed in the following sections. We visualize the predicted host-pathogen PPIN consisting of these 92 *H. sapiens-M. tuberculosis *H37Rv PPIs using Cytoscape [22] in Figure 1. The orange dots are *M. tuberculosis *H37Rv proteins, while the blue dots are *H. sapiens *proteins. The predicted *H. sapiens-M. tuberculosis *H37Rv PPI dataset can be found in the Additional Files 1. From Figure 1 we can observe that, like many host-pathogen PPINs, the pathogen proteins tend to be hubs in host-pathogen PPIN.

### Prediction of intra-species PPIs

Currently no large-scale high-quality *H. sapiens-M. tuberculosis *H37Rv dataset is available. So we can not directly assess the performance of our stringent DDI-based approach in the inter-species host-pathogen system. Reluctantly, we turn to the intra-species system for the assessments. We predict intra-species *H. sapiens *PPIs using the stringent and the conventional DDI-based approaches. Altogether 839 *H. sapiens *PPIs are predicted by the stringent DDI-based approach. In contrast, 724185 *H. sapiens *PPIs are predicted by the conventional DDI-based approach. Just from the number of PPIs predicted by two approaches the differences are obvious. Our stringent DDI-based approach relies on very high sequence similarity to the template domain instances and stands on the stringent domain instances to make the prediction. Therefore only a small amount of PPIs are predicted. And the small number of structurally resolved template interacting domain instances also limits the number of PPIs we can predict using our stringent DDI-based approach. Whereas the conventional DDI-based approach derives the possible interacting domain information from known PPI datasets(which can be abundant for some species), and treats all domain instances annotated under the same domain ID as the same. So a large number of PPIs can be predicted by the conventional DDI-based approach. We compare the performance of our stringent DDI-based approach and the conventional DDI-based approach based on gold standard PPI datasets and percentage of PPIs having coherent informative GO terms.

### Assessment based on gold standard *H. sapiens *PPIs

We collect the known *H. sapiens *physical PPI dataset from MINT [13], BioGRID [14], and IntAct [15] as our gold standard PPI dataset to assess the *H. sapiens *PPIs predicted by the stringent and the conventional DDI-based approaches. We calculate and plot the precision-recall curve of the stringent and the conventional DDI-based approaches; see Figure 2. From the plots we can see both of the prediction approaches achieve better precision when the threshold increases. This shows that the scoring strategies adopted by both prediction approaches in calculating the "interaction strength" are valid in telling the likelihood of predicted PPIs being real. From the precision-recall curves, one can clearly tell that overall the stringent DDI-based approach consistently predicts PPIs with much higher precision than that of the conventional DDI-based approach; see Figure 2. As the conventional DDI-based approach makes a large number of predictions, it has higher recall. The precision-recall curve shows that our stringent DDI-based approach can only predict small amount of PPIs but with much higher accuracy than the conventional approach. As the two approaches predict very different number of PPIs, we also choose some special points to compare the performance of the two prediction approaches, see Table 1. We can see that when our stringent DDI-based approach predicts 839 *H. sapiens *PPIs, 82 of which overlap with the gold standard; when the conventional DDI-based approach predicts 885 *H. sapiens *PPIs, only 11 of which overlap with the gold standard. Our stringent DDI-based approach has to predict 839 *H. sapiens *PPIs in order to have 82 *H. sapiens *PPIs overlapping with the gold standard. The conventional DDI-based approach has to predict 3085 *H. sapiens *PPIs in order to have 81 *H. sapiens *PPIs overlapping with the gold standard; see Table 1. All these assessments using the gold standard *H. sapiens *PPIs clearly show that our stringent DDI-based approach is more stringent and has better performance than that of the conventional DDI-based approach.

### Assessment based on coherent informative GO annotation of predicted *H. sapiens *PPIs

To further compare the performance of the stringent and the conventional DDI-based approaches, we calculate the percentage of PPIs that have coherent informative GO terms. From Figure 3 and Figure 4, the overall percentage of PPIs having coherent informative GO terms reveals that both approaches work well--as moving towards to a higher threshold (smaller number of top PPIs) leads to a higher percentage of PPIs having coherent informative GO terms. As shown in Figure 3, the PPI dataset predicted by our stringent DDI-based approach starts with high percentage of PPIs having coherent informative GO terms; this indicates overall good performance as the PPI dataset predicted by our stringent DDI-based approach has low noise level and high quality. In contrast, the PPI dataset predicted by the conventional DDI-based approach does not show as good performance as the stringent DDI-based approach in terms of the overall percentage of PPIs having coherent informative GO terms--the PPI dataset predicted by the conventional DDI-based approach starts with a low percentage of PPIs having coherent informative GO terms, especially very low percentage of cellular compartment (CC) terms and biological process (BP) terms; this indicates that the PPI dataset predicted by the conventional DDI-based approach has high noise and the quality is not good. As the PPI datasets predicted by the two approaches are very different in the number of predicted PPIs, it may not be a sufficient assessment seeing only overall plots of percentage of PPIs having coherent informative GO terms. Therefore, we focus on the top 839 PPIs respectively predicted by the stringent and the conventional DDI-based approaches and plot their percentage of PPIs having coherent informative GO terms in Figure 5. We can clearly observe that PPIs predicted by the stringent DDI-based approach have consistently higher percentage of coherent informative CC and BP terms; see Figure 5. The percentage of PPIs that have coherent informative GO terms may also be influenced by the number of GO terms that are annotated to the proteins in the PPI datasets. So we summarize the number of GO terms that are annotated to proteins in all 839 PPIs predicted by the stringent DDI-based approach, and proteins in all 724185 PPIs and the top 839 PPIs predicted by the conventional DDI-based approach in Table 2. This table shows that although a high percentage of the PPIs predicted at a high threshold by the conventional DDI-based approach has coherent informative GO terms, this may be due the fact that these top 839 PPIs are annotated with very few distinct GO terms. Even with such a smaller number of informative GO terms we can see that the percentage of PPIs predicted by the conventional DDI-based approach having coherent informative GO terms is still consistently lower than the stringent DDI-based approach; this strongly supports the conclusion that the stringent DDI-based approach has a much better performance than that of the conventional DDI-based approach in predicting reliable PPIs.

### Cellular compartment distribution of *H. sapiens *proteins targeted by predicted host-pathogen PPIs.

The cellular compartment distribution of the *H. sapiens *proteins targeted by the host-pathogen PPIs predicted by our stringent DDI-based approach is an important indicator of the performance of the prediction approach and the quality of the *H. sapiens-M. tuberculosis *H37Rv PPIs predicted. If the targeted *H. sapiens *proteins are mostly located in cellular compartments having a close relationship with pathogen infection then the predicted results are more convincing. We identify the informative CC terms in *H. sapiens *proteins. Then we calculate the number and percentage of proteins in the datasets that have been annotated with each of the informative CC terms. Then we plot the located informative CC terms for the targeted *H. sapiens *proteins by the stringent DDI-based approach in Figure 6, with detail statistics given in Table 1.

Many of the host-pathogen PPIs predicted by the stringent DDI-based approach target *H. sapiens *proteins located in very relevant cellular compartments. *M. tuberculosis *H37Rv infection has a close relationship with mitochondria activities and function and induces quantitatively distinct changes in the mitochondrial proteome [25]. Ultrastructural changes in the mitochondria and mitochondrial clustering are also observed in the *M. tuberculosis *H37Rv infected cells [25]. The augmentation of mitochondrial activity by *M. tuberculosis *H37Rv enables manipulation of host cellular mechanisms to inhibit apoptosis and ensure fortification against anti-microbial pathways [25]. Therefore mitochondrial matrix(GO:0005759), mitochondrial outer membrane(GO:0005741) and proton-transporting two-sector ATPase complex(GO:0016469), are relevant to *M. tuberculosis *H37Rv infection.

*H. sapiens *proteins located at flagellum (GO:0019861) have much higher chance of interacting with *M. tuberculosis *H37Rv during infection as proteins located at flagellum are the first set of proteins that *M. tuberculosis *H37Rv comes across before invading the cell.

The CC term peroxisomal part(GO:0044439) is also strongly related to *M. tuberculosis *infection. It is found that the interaction between the mycobacterial phagosome and the endoplasmic reticulum leads to proteasome degradation and MHC class I presentation of *M. tuberculosis *antigens.

Focal adhesion(GO:0005925) is also closely interconnected to the *M. tuberculosis *infection process. In many bacterial pathogens, protein tyrosine phosphatases (PTPases) are essential for dephosphorylating host focal adhesion proteins and focal adhesion kinase. This dephosphorylation leads to destabilization of focal adhesions involved in the internalization of bacterial pathogens by eukaryotic cells [26,27]. Therefore the proteins located at "Focal adhesion" compartment are very important target for *M. tuberculosis *infection of host. This strongly supports the validity of the prediction results of our stringent DDI-based approach.

The cellular compartment lamellipodium(GO:0030027) also supports the validity of our prediction results. It has been reported that host cell's actin filament network is interfered by pathogenic species of mycobateria [28-30]. A more recent study shows that *M. tuberculosis *affects actin polymerisation [31].

The CC term nucleolus(GO:0005730) may also be related to *M. tuberculosis *infection, as *M. tuberculosis *infection of human macrophages blocks several responses to IFN-*γ*. The inhibitory effect of *M. tuberculosis *is directed at the transcription of IFN-*γ*-responsive genes [32]. Several studies show that *M. tuberculosis *and its purified protein derivative induced HIV LTR primarily through transcriptional activation [33].

The cellular compartment distribution analysis of the *H. sapiens *proteins targeted by host-pathogen PPIs strongly supports the validity of the PPI dataset predicted by our stringent DDI-based approach.

### Functional enrichment analysis of proteins involved in host-pathogen PPIs

Functional enrichment analysis points out the possible functional relevance of *H. sapiens *proteins involved in the *H. sapiens-M. tuberculosis *H37Rv PPIN predicted by the stringent DDI-based approaches. The representative result--the most significantly enriched level 5 MF GO terms--is given in Table 4.

Most of the significantly enriched functional categories are strongly related to *M. tuberculosis *H37Rv infection, including adenyl ribonucleotide binding(GO:0032559), ATPase activity, coupled to transmembrane movement of substances (GO:0042626), P-P-bond-hydrolysis-driven transmembrane transporter activity(GO:0015405), ATPase activity, coupled to transmembrane movement of ions(GO:0042625), long-chain-acyl-CoA dehydrogenase activity(GO:0004466), NADPH:quinone reductase activity(GO:0003960), NADPH binding(GO:0070402), retinol dehydrogenase activity(GO:0004745), retinol binding(GO:0019841), and MHC class I protein binding(GO:0042288).

As described above, *M. tuberculosis *H37Rv infection is closely related to the mitochondria. Therefore all those MF terms closely related to mitochondria are relevant to *M. tuberculosis *H37Rv infection; the relevant GO terms include ATPase activity, coupled to transmembrane movement of substances (GO:0042626), P-P-bond-hydrolysis-driven transmembrane transporter activity(GO:0015405), ATPase activity, coupled to transmembrane movement of ions(GO:0042625), NADPH:quinone reductase activity(GO:0003960), NADPH binding(GO:0070402).

MHC class I protein binding(GO:0042288) is a strongly immune-related term which is also very relevant to *M. tuberculosis *H37Rv infection. Proteins enriched in this term play an important role in presenting *M. tuberculosis *antigens, which is essential for the immune response to this pathogen.

The long-chain-acyl-CoA dehydrogenase activity(GO:0004466) is a fatty acid-related term which is very relevant to *M. tuberculosis *H37Rv infection. Fatty acids and cholesterol appear to be the favored nutrients for *M. tuberculosis *inside *H. sapiens *cells [34]. The breakdown of fatty acids and cholesterol can generate propionyl-CoA, which gives rise to potentially toxic intermediates [34]. Through the methylcitrate cycle, the methylmalonyl pathway, or incorporation of the propionyl-CoA into methyl-branched lipids in the cell wall, *M. tuberculosis *expands the acetyl-CoA pool and alleviates the pressure from propionyl-CoA [34].

This functional enrichment analysis shows that our stringent DDI-based approach is accurate and has merits in identifying possible *H. sapiens *proteins that are involved in *H. sapiens-M. tuberculosis *H37Rv PPIs.

### Pathway enrichment analysis of proteins involved in host-pathogen PPIs

Pathway enrichment analysis of the proteins involved in host-pathogen PPIN can provide rich information on the functional relevance of (both the host and pathogen) proteins involved in the host-pathogen PPIN. The analysis should show that the host proteins involved in host-pathogen interactions is a set of proteins that have functional correlation to pathways relevant to the pathogen's infection. Indeed *H. sapiens *proteins involved in the *H. sapiens-M. tuberculosis *H37Rv PPIN predicted by the stringent DDI-based approach are mostly enriched in the pathways are closely relevant to *M. tuberculosis *infection; see Table 5. For example, "Fatty Acid Metabolism", "Fatty Acid Beta Oxidation", and "Glycolysis and Gluconeogenesis" are closely related to *M. tuberculosis *infection as fatty acids are one of the favored nutrients for *M. tuberculosis *inside *H. sapiens *cells [34]. *M. tuberculosis *is able to grow on a variety of carbon sources, but mounting evidence has implicated fatty acids as the major source of carbon and energy for *M. tuberculosis *during infection [35]. And *M. tuberculosis *switches its carbon source from sugars to fatty acids during the persistent phase of infection [36]. Biosynthesis of sugars from intermediates of the tricarboxylic acid cycle is essential for growth [35]. The pathways "Metabolic Pathways", "Valine, Leucine and Isoleucine Degradation", "2-Oxobutanoate Degradation I", and "Ethanol Degradation II (cytosol)" maybe also be very related to *M. tuberculosis *infection as they are closely involved with intermediates of the tricarboxylic acid cycle which is essential for the growth of *M. tuberculosis *[35]. And they may also contribute to the carbon flow of *M. tuberculosis *metabolism inside the human cell.

*M. tuberculosis *H37Rv proteins involved in the *H. sapiens-M. tuberculosis *H37Rv PPIN predicted by the stringent DDI-based approach are significantly enriched in the "Fatty Acid *β *oxidation I" pathway, see Table 6. This strongly supports the validity of our prediction results. As discussed above, fatty acids are the major source of carbon and energy for *M. tuberculosis *during infection [35], and pathways involved with fatty acids metabolism strongly indicate association with the infection state of *M. tuberculosis *H37Rv. It is found that when the pathogen's acyl-coenzyme A synthetase gene is disrupted, infected mice survive significantly longer than those infected with the wild type, thus suggesting attenuation of the mutated pathogen. In fact the pathogen never attains the plateau phase of infection in mouse lungs when pathogen's acyl-coenzyme A synthetase gene is disrupted [37]. *M. tuberculosis *fatty acyl-coenzyme A synthetase gene may serve to recycle mycolic acids for the long-term survival of the tubercle bacilli [37]. Carbon rerouting is marked by a switch from metabolic pathways generating energy and biosynthetic precursors in growing bacilli to pathways for storage compound synthesis during growth arrest [36]. This analysis result is in accord with the above cellular compartment distribution, functional enrichment analysis.

All the results support the validity of the *H. sapiens-M. tuberculosis *H37Rv PPIs predicted by our stringent DDI-based approach. Therefore the prediction results from our stringent DDI-based approach can serve as a reliable reference of PPIs between *H. sapiens *and *M. tuberculosis *H37Rv.

### Analysis of domain properties of proteins involved in host-pathogen PPIs

We compare two domain properties of both *H. sapiens *and *M. tuberculosis *H37Rv proteins in the predicted *H sapiens-M. tuberculosis *H37Rv PPIN and their own intra-species PPIN. We also conduct a similar analysis on *H. sapiens *proteins involved in the gold standard *H. sapiens*-HIV PPIN [19] as a control experiment. Table 7 provides summary results from the analysis of *H. sapiens *and *M. tuberculosis *H37Rv proteins. It is obvious that *H. sapiens *proteins targeted by the predicted *H. sapiens-M. tuberculosis *H37Rv PPIN show properties very similar to those *H. sapiens *proteins targeted by the gold standard *H. sapiens*-HIV PPIN [19]. This also supports the validity of our prediction results to some extent.

Both in the predicted *H. sapiens-M. tuberculosis *H37Rv PPIN and in the gold standard *H. sapiens*-HIV PPIN, *H. sapiens *proteins tend to have more domains and those domains tend to have higher degrees than those proteins in the intra-species *H. sapiens *PPIN.

The discoveries found by analyzing domain properties may be helpful in illuminating the basic mechanisms of how the host and pathogen proteins interact with each other, and may be useful in assessing the predicted host-pathogen PPIN.

## Discussion

### Sequence similarity between domain instances in DDI-based prediction

Comparing with conventional DDI-based approaches, our stringent DDI-based approach emphasizes the importance of domain instances in inferring interactions from template DDIs. While this emphasis on stringent sequence similarity between template and query domain instances in transferring interaction results in significant improvement on prediction performance, it also draws attention to the large sequence variation among domain instances which may limit conventional DDI-based approaches.

It is also noteworthy that many new prediction algorithms based on the stringent alignment of domain instances can be proposed to predict possible intra- and inter-species PPIs.

### Pros and cons of DDI-based prediction

The advantages of our stringent DDI-based approach have been discussed above, as it can predict more accurate PPIs on a small scale. The possible limitation of this approach is the lack of large-scale high-quality structurally-resolved DDIs. However, it is reasonable to expect more protein complex structures will be resolved, and the effectiveness of our stringent DDI-based approach will consequently be significantly strengthened.

Producing only a small amount of PPIs does not distract us from the merits of our stringent DDI-based approach, because the small number of highly accurate PPIs may already be more valuable than a huge amount of PPIs with a substantial fraction of noise. Highly accurate predicted PPIs, even though small in size, are usually very welcomed in experimental research, as they are a much more valuable reference for experimental verification than large datasets with high noise.

Accurate sequence alignment among domain instances are much more computationally expensive than the conventional DDI-based approach. This may limit the application of our stringent DDI-based approach to large-scale prediction of PPIs across many host-pathogen systems.

## Conclusion

In this work, we have proposed a stringent DDI-based prediction approach based on high sequence similarity between template domain instances and query domain instances. The assessment based on gold-standard *H. sapiens *PPIs and informative GO annotation shows that the stringent DDI-based approach performs better than the conventional DDI-based approach. We have also predicted a small set of accurate *H. sapiens-M. tuberculosis *H37Rv PPIs. Through cellular compartment distribution, functional enrichment, and pathway enrichment analysis, we have demonstrated that this small set of accurate *H. sapiens-M. tuberculosis *H37Rv PPIs is valid and closely corresponds to *M. tuberculosis *H37Rv infection. This dataset of *H. sapiens-M. tuberculosis *H37Rv PPIs can be used for a variety of related studies as an important reference.

## Competing interests

The authors declare that they have no competing interests.

## Authors' contributions

This work was jointly conceived, planned, and written up by Limsoon Wong and Hufeng Zhou. The analytical experiments were performed by Hufeng Zhou. The DDI scoring strategy was proposed and implemented by Willy Hugo and Javad Rezaie. The physical PPI datasets were collected and prepared by Chern-Han Yong. The visualization of PPIN was done by Michal Wozniak. The functional analysis was proposed, suggested and implemented by Hufeng Zhou, Shangzhi Gao, Jingjing Jin and Mengyuan Fan. The statistical test was jointly implemented by Shangzhi Gao and Hufeng Zhou.

## Further information

Interacting domain instances and structural information from 3did can be downloaded from: http://compbio.ddns.comp.nus.edu.sg/~zhouhufeng/Research/DDIbased/data/.

## Supplementary Material

Additional file 1 Predicted *H. sapiens-M. tuberculosis *H37Rv PPI datasets. We predicted *H. sapiens-M. tuberculosis *H37Rv PPIs using our stringent DDI-based prediction approach. The predicted PPI data are recorded in simple text format in additional file 1. Click here for file

## Declaration

Funding for the publication of this paper is provided by Wong's KITHCT chair professorship.

This article has been published as part of *BMC Systems Biology *Volume 7 Supplement 6, 2013: Selected articles from the 24th International Conference on Genome Informatics (GIW2013). The full contents of the supplement are available online at http://www.biomedcentral.com/bmcsystbiol/supplements/7/S6.

## References

[B1] Butler D New fronts in an old war Nature 2000 7 6797 670 672 10.1038/35021291 10963570

[B2] Koul A Herget T Klebl B Ullrich A Interplay between mycobacteria and host signalling pathways Nature Reviews Microbiology 2004 7 3 189 202 10.1038/nrmicro840 15083155

[B3] Hestvik A Hmama Z Av-Gay Y Mycobacterial manipulation of the host cell FEMS Microbiology Reviews 2006 7 5 1041 1050 10.1016/j.femsre.2005.04.013 16040149

[B4] Global Tuberculosis Programme WHO Global Tuberculosis Control: WHO Report 2010 Global Tuberculosis Programme, World Health Organization

[B5] Zhou H Jin J Wong L Progress in computational studies of host-pathogen interactions J Bioinform Comput Biol 2013 7 2 1230001 10.1142/S0219720012300018 23600809

[B6] Edgar RC MUSCLE: Multiple sequence alignment with high accuracy and high throughput Nucleic Acids Research 2004 7 5 1792 1797 10.1093/nar/gkh340 15034147 PMC390337

[B7] Stein A Céol A Aloy P 3did: Identification and classification of domain-based interactions of known three-dimensional structure Nucleic Acids Research 2011 7 suppl 1 D718 D723 20965963 10.1093/nar/gkq962 PMC3013799

[B8] Dyer MD Murali TM Sobral BW Computational prediction of host-pathogen protein-protein inter-actions Bioinformatics 2007 7 13 i159 i166 10.1093/bioinformatics/btm208 17646292

[B9] The UniProt Consortium Reorganizing the protein space at the Universal Protein Resource (UniProt) Nucleic Acids Research 2012 7 D1 D71 D75 22102590 10.1093/nar/gkr981 PMC3245120

[B10] Hunter S Jones P Mitchell A Apweiler R Attwood TK Bateman A Bernard T Binns D Bork P Burge S et al. InterPro in 2011: New developments in the family and domain prediction database Nucleic Acids Research 2012 7 D1 D306 D312 10.1093/nar/gkr948 22096229 PMC3245097

[B11] Henikoff S Henikoff JG Amino acid substitution matrices from protein blocks Proceedings of the National Academy of Sciences USA 1992 7 22 10915 10919 10.1073/pnas.89.22.10915 PMC50453 1438297

[B12] Sprinzak E Margalit H Correlated sequence-signatures as markers of protein-protein interaction Journal of Molecular Biology 2001 7 4 681 692 10.1006/jmbi.2001.4920 11518523

[B13] Zanzoni A Montecchi-Palazzi L Quondam M Ausiello G Helmer-Citterich M Cesareni G MINT: a Molecular INTeraction database FEBS Letters 2002 7 135 140 10.1016/S0014-5793(01)03293-8 11911893

[B14] Stark C Breitkreutz B Chatr-Aryamontri A Boucher L Oughtred R Livstone M Nixon J Van Auken K Wang X Shi X et al. The BioGRID interaction database: 2011 update Nucleic Acids Research 2011 7 suppl 1 D698 D704 21071413 10.1093/nar/gkq1116 PMC3013707

[B15] Hermjakob H Montecchi-Palazzi L Lewington C Mudali S Kerrien S Orchard S Vingron M Roechert B Roepstorff P Valencia A IntAct: An open source molecular interaction database Nucleic Acids Research 2004 7 suppl 1 D452 D455 14681455 10.1093/nar/gkh052 PMC308786

[B16] Zhou H Wong L Comparative analysis and assessment of *M. tuberculosis *H37Rv protein-protein interaction datasets BMC Genomics 2011 7 Suppl 3 S20 10.1186/1471-2164-12-S3-S20 22369691 PMC3333180

[B17] Dennis G Jr Sherman B Hosack D Yang J Gao W Lane H Lempicki R DAVID: Database for annotation, visualization, and integrated discovery Genome Biology 2003 7 5 P3 10.1186/gb-2003-4-5-p3 12734009 PMC193660

[B18] Zhou H Jin J Zhang H Bo Y Wozniak M Wong L IntPath--an integrated pathway gene relationship database for model organisms and important pathogens BMC System Bio 2012 7 Suppl 2 S2 10.1186/1752-0509-6-S2-S2 PMC3521174 23282057

[B19] Fu W Sanders-Beer B Katz K Maglott D Pruitt K Ptak R Human immunodeficiency virus type 1, human protein interaction database at NCBI Nucleic Acids Research 2009 7 suppl 1 D417 D422 18927109 10.1093/nar/gkn708 PMC2686594

[B20] Eddy S Accelerated profile HMM searches PLoS Computational Biology 2011 7 10 e1002195 10.1371/journal.pcbi.1002195 22039361 PMC3197634

[B21] Bateman A Coin L Durbin R Finn R Hollich V Griffiths-Jones S Khanna A Marshall M Moxon S Sonnhammer E The Pfam protein families database Nucleic acids research 2004 7 suppl 1 D138 D141 14681378 10.1093/nar/gkh121 PMC308855

[B22] Smoot M Ono K Ruscheinski J Wang P Ideker T Cytoscape 2.8: new features for data integration and network visualization Bioinformatics 2011 7 3 431 432 10.1093/bioinformatics/btq675 21149340 PMC3031041

[B23] Quevillon E Silventoinen V Pillai S Harte N Mulder N Apweiler R Lopez R InterProScan: Protein domains identifier Nucleic Acids Research 2005 7 suppl 2 W116 W120 15980438 10.1093/nar/gki442 PMC1160203

[B24] Yellaboina S Tasneem A Zaykin D Raghavachari B Jothi R DOMINE: A comprehensive collection of known and predicted domain-domain interactions Nucleic Acids Research 2011 7 suppl 1 D730 D735 21113022 10.1093/nar/gkq1229 PMC3013741

[B25] Jamwal S Midha MK Verma HN Basu A Rao KV Manivel V Characterizing virulence-specific perturbations in the mitochondrial function of macrophages infected with mycobacterium tuberculosis Scientific Reports 2013 7 1328 23435464 10.1038/srep01328 PMC3580321

[B26] Persson C Carballeira N Wolf-Watz H Fällman M The PTPase YopH inhibits uptake of Yersinia, tyrosine phosphorylation of p130Cas and FAK, and the associated accumulation of these proteins in peripheral focal adhesions The EMBO Journal 1997 7 9 2307 2318 10.1093/emboj/16.9.2307 9171345 PMC1169832

[B27] Black D Bliska J Identification of p130Cas as a substrate of Yersinia YopH (Yop51), a bacterial protein tyrosine phosphatase that translocates into mammalian cells and targets focal adhesions The EMBO Journal 1997 7 10 2730 2744 10.1093/emboj/16.10.2730 9184219 PMC1169883

[B28] Guérin I de Chastellier C Pathogenic mycobacteria disrupt the macrophage actin filament network Infection and Immunity 2000 7 5 2655 2662 10.1128/IAI.68.5.2655-2662.2000 10768957 PMC97472

[B29] Guérin I de Chastellier C Disruption of the actin filament network affects delivery of endocytic contents marker to phagosomes with early endosome characteristics: the case of phagosomes with pathogenic mycobacteria European Journal of Cell Biology 2000 7 10 735 749 10.1078/0171-9335-00092 11089922

[B30] Anes E Kühnel M Bos E Moniz-Pereira J Habermann A Griffiths G Selected lipids activate phagosome actin assembly and maturation resulting in killing of pathogenic mycobacteria Nature cell biology 2003 7 9 793 802 10.1038/ncb1036 12942085

[B31] Esposito C Marasco D Delogu G Pedone E Berisio R Heparin-binding hemagglutinin HBHA from *My-cobacterium tuberculosis *affects actin polymerisation Biochemical and Biophysical Research Communications 2011 7 2 339 344 10.1016/j.bbrc.2011.05.159 21672524

[B32] Ting L Kim A Cattamanchi A Ernst J *Mycobacterium tuberculosis *inhibits IFN-*γ *transcriptional responses without inhibiting activation of STAT1 The Journal of Immunology 1999 7 7 3898 3906 10490990

[B33] Toossi Z Xia L Wu M Salvekar A Transcriptional activation of HIV by *Mycobacterium tuberculosis *in human monocytes Clinical and Experimental Immunology 1999 7 2 324 330 10.1046/j.1365-2249.1999.00952.x 10444265 PMC1905327

[B34] Lee W VanderVen BC Fahey RJ Russell DG Intracellular *Mycobacterium tuberculosis *exploits host-derived fatty acids to limit metabolic stress Journal of Biological Chemistry 2013 7 10 6788 6800 10.1074/jbc.M112.445056 23306194 PMC3591590

[B35] Marrero J Rhee KY Schnappinger D Pethe K Ehrt S Gluconeogenic carbon flow of tricarboxylic acid cycle intermediates is critical for *Mycobacterium tuberculosis *to establish and maintain infection Proceedings of the National Academy of Sciences USA 2010 7 21 9819 9824 10.1073/pnas.1000715107 PMC2906907 20439709

[B36] Shi L Sohaskey CD Pfeiffer C Datta P Parks M McFadden J North RJ Gennaro ML Carbon flux rerouting during *Mycobacterium tuberculosis *growth arrest Molecular Microbiology 2010 7 5 1199 1215 10.1111/j.1365-2958.2010.07399.x 21091505 PMC3072047

[B37] Dunphy KY Senaratne RH Masuzawa M Kendall LV Riley LW Attenuation of *Mycobacterium tuberculosis *functionally disrupted in a fatty acyl-coenzyme A synthetase gene fadD5 Journal of Infectious Diseases 2010 7 8 1232 1239 10.1086/651452 20214478 PMC3225055

